# Unlocking the PACS DICOM Domain for its Use in Clinical Research Data Warehouses

**DOI:** 10.1007/s10278-020-00334-0

**Published:** 2020-04-20

**Authors:** Mathias Kaspar, Leon Liman, Maximilian Ertl, Georg Fette, Lea Katharina Seidlmayer, Laura Schreiber, Frank Puppe, Stefan Störk

**Affiliations:** 1 Comprehensive Heart Failure Center and Department of Internal Medicine I, Würzburg University Hospital, Würzburg, Germany; 2 Department of Health Services Research, Carl von Ossietzky University of Oldenburg, Oldenburg, Germany; 3 Chair of Computer Science VI, University of Würzburg, Würzburg, Germany; 4 Service Center Medical Informatics, Würzburg University Hospital, Würzburg, Germany

**Keywords:** Clinical data warehouse, Medical images, System integration, Secondary data usage

## Abstract

Clinical Data Warehouses (DWHs) are used to provide researchers with simplified access to pseudonymized and homogenized clinical routine data from multiple primary systems. Experience with the integration of imaging and metadata from picture archiving and communication systems (PACS), however, is rare. Our goal was therefore to analyze the viability of integrating a production PACS with a research DWH to enable DWH queries combining clinical and medical imaging metadata and to enable the DWH to display and download images ad hoc. We developed an application interface that enables to query the production PACS of a large hospital from a clinical research DWH containing pseudonymized data. We evaluated the performance of bulk extracting metadata from the PACS to the DWH and the performance of retrieving images ad hoc from the PACS for display and download within the DWH. We integrated the system into the query interface of our DWH and used it successfully in four use cases. The bulk extraction of imaging metadata required a median (quartiles) time of 0.09 (0.03–2.25) to 12.52 (4.11–37.30) seconds for a median (quartiles) number of 10 (3–29) to 103 (8–693) images per patient, depending on the extraction approach. The ad hoc image retrieval from the PACS required a median (quartiles) of 2.57 (2.57–2.79) seconds per image for the download, but 5.55 (4.91–6.06) seconds to display the first and 40.77 (38.60–41.63) seconds to display all images using the pure web-based viewer. A full integration of a production PACS with a research DWH is viable and enables various use cases in research. While the extraction of basic metadata from all images can be done with reasonable effort, the extraction of all metadata seems to be more appropriate for subgroups.

**Electronic supplementary material:**

The online version of this article (10.1007/s10278-020-00334-0) contains supplementary material, which is available to authorized users.

## Introduction

Clinical Data Warehouses (DWHs) are used to provide researchers with simplified access to pseudonymized clinical routine data from multiple primary systems. DWHs can provide data from various domains that are available in structured form, semi-structured form, or narrative texts. Most available data might originate from domains that are already at least semi-structured (e.g., for accounting reasons), such as administrative data, diagnoses, procedures, diagnosis-related groups (DRGs), and laboratory values. From radiology, however, many clinical DWHs only integrate radiology reports [1–3]. The imaging data itself, stored in picture archiving and communication systems (PACS), are usually not as comprehensively integrated with DWHs as other data sources, even though they are also of great interest for clinical and imaging research [4–8] as well as for education [9]. A relevant factor that has to be considered is the large amount of data stored in a PACS. This makes a complete copy less feasible and creates a trade-off between a direct mass access for research and the provision of sufficient performance for the primary clinical use.

### Related Work

Clinical research requires dedicated data storage to build controlled data sets for defined study groups. Since imaging data have other properties than most structured clinical data (e.g., large binary data versus structured numeric and textual data), they are often stored and used separately. Consequently, dedicated complementary research PACS were developed allowing to store, query, view, and analyze imaging data [10], e.g., XNAT [11] or DCM4CHEE [12].

Research projects that focus on imaging utilize specialized imaging DWHs to enable extensive querying of DICOM metadata in separate stores [4, 13] and to store various imaging-related data structures and images [14], without focus on clinical data. Cohen et al. [15] described the integration and its utilization of DICOM header data from a PACS via HL7-XML into an electronic medical record. Langer et al. developed a DICOM DWH that takes DICOM images from a PACS or the modality via the hospital’s transactional process, stores and provides their metadata for comprehensive analysis, with a focus on the harmonization of header data of different vendors [16, 17]. Other projects developed systems that can be used to query, extract, or integrate DICOM images directly from a production PACS to create subsets for specific research projects [13, 18, 19].

The systems described above specialize in handling imaging-related data, partially focus on just providing header data for imaging research, and rather work on subgroups of the total hospital patients. If used with a clinical DWH, they would require a separate handling, likely including separate pseudonymization steps. We have not found a report on a fully integrated solution of imaging and clinical data. If DICOM files were integrated with clinical DWHs, they store linked DICOM files separately, e.g., on a dedicated imaging system [20–22] or a special PACS [23]. They did not evaluate the integration of a full production PACS. Murphy et al. [24] described the most elaborate system we have found. They developed a module for i2b2 that allows for an interactive image retrieval from multiple PACS, based on a patient selection made in preceding steps. They did not integrate results retrieved from the PACS directly into a DWH but provided tools to send images to a connected study-specific image store, e.g., an XNAT instance.

### Objectives

Various projects provide data from PACS for research by enabling the query and retrieval of images from the PACS or the storage of images in separate systems, but do not comprehensively integrate clinical and PACS data for combined use with all patients of a hospital. Thus, our goal was to analyze the viability of integrating a fully production PACS with a research DWH in order:

1. to enable queries combining clinical data and DICOM header data for all patients of a large hospital,
2. to enable the immediate view of DICOM images from the PACS for the queried patients in a comfortable GUI, and
3. to download DICOM images together with attributes from the DWH for further processing.

By developing such a system, we intended to ease access to research that requires information on medical images, their metadata, and clinical data. We focused on enabling the following use cases at our institution:

1. query the DWH using combinations of DICOM metadata and clinical data to discover patient cohorts,
2. extract imaging data for clinical studies,
3. query and extract imaging and clinical data for the training of neural networks, and
4. extract common and special DICOM metadata from the PACS for analysis.

## Methods

### Clinical Data Warehouse

At the Würzburg University Hospital, a clinical DWH has been implemented that provides homogenized and pseudonymized data of all patients and cases from multiple subsystems [25–27]. Routine data is included as structured data (e.g., patient demographics, diagnoses, procedures, electronic patient file from wards, and laboratory values), semi-structured data (e.g., forms for procedures like coronary angiography and study marker), and narrative text (e.g., discharge letters and echocardiography reports). Among others, the DWH query interface allows to query for (non-)existence and conditions in numeric and date time values. It also provides an ad hoc information extraction functionality to search and extract words in narrative texts using regular expressions [28].

### Evaluation

We developed a PACS-to-DWH (P2D) application interface that enables to query the production PACS of our hospital using the DICOM standard from our clinical research DWH containing pseudonymized data. The system was used in several use cases to show the overall viability of the system, which is described in the results.

The viability of the combined query of hospital and PACS data is shown in the first use case. Its functionality is based on the bulk extraction of DICOM metadata from the PACS into the DWH database, from where the data can be queried with the usual DWH query system. The performance of the DWH query system was neither tested nor evaluated in the work presented here.

The amount of data within a PACS can be very large, which makes a complete export not viable. We therefore measured the extraction performance of three scenarios from the production PACS and extrapolated the figures in order to estimate times required for a whole hospital. The test data set consisted of 1000 patients, who were evenly distributed among all DWH patients (sorted by date) with an existing radiology report. The test was repeated with the open source DICOM server Orthanc^1^ in its default configuration and prepared with extracted and anonymized data of the first 20 patients. However, the Orthanc deployment cannot be fully compared with the resources of a production PACS and shall only provide an orientation for a comparison. Number of images, times, and data rates are presented as mean, median, quartiles, maximum, and minimum values.

The functionality to display images of patients on demand is shown in screenshots. It is based on the ad hoc PACS query system which provides a real-time PACS query functionality and is typically used from within the DWH query interface. Therefore, we performed measurements for its evaluation during real use in the DWH user interface (10 iterations each at different times of the day). The Firefox Network Monitor was used to record detailed data.

The ad hoc PACS query is also the basis for the image download, which is evaluated in the same way as displaying images. Use case three further describes an example of extracting combinations of imaging and clinical data.

All data collected during the tests were analyzed using R. The performance of the P2D interface was tested in the production environment with a single thread querying the PACS without throttling. The DWH system and Orthanc are deployed on a computer with 2 Xeon E5-2643 and 512 GB RAM. The P2D interface is deployed on a computer with a Xeon E5-2643 and 384 GB RAM. Client computers are virtual machines with a Xeon 6152 and 4 GB RAM.

### System Design

**Fig. 1:**
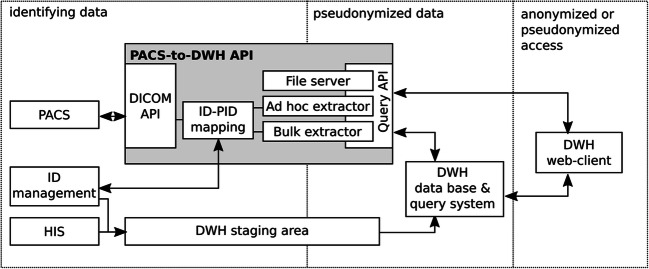
Architecture of the DWH including the PACS-to-DWH (P2D) interface application. The P2D interface (gray background) connects to the PACS, to the identity (ID) management system, to the DWH database (via the metadata extractor), and to the DWH web-client (via the ad hoc query system)

The PACS-to-DWH (P2D) interface was developed as a stand-alone server application that can be integrated into a DWH system. It is based on a PACS and an identity management system. Figure 1 illustrates the structure of our DWH environment including the P2D interface. Towards a PACS, the P2D interface provides a standard DICOM query/retrieve interface using the identifiers known to the PACS. Towards the DWH, the P2D interface provides a REST-style interface that takes queries using the identifiers known to the DWH. Another interface supports the connection to an identity management system that is required to translate between the identifiers.

The P2D DICOM interface is required to query/retrieve DICOM data sets from a PACS using standard DICOM Message Exchange provided by the DICOM Toolkit (DCMTK).^2^ It enables the P2D interface to query the PACS for DICOM data sets (i.e., DICOM images). The usage of DICOM C-FIND allows to retrieve a restricted set of DICOM headers.^3^ The usage of DICOM C-MOVE allows to retrieve the whole data sets (including pixel data), which is also the approach of choice if only a single special tag is required. Internally, the interface provides methods to find and download data sets by using the following parameters: time ranges, patient identifiers, Instance UIDs, accession numbers, and modalities. All DICOM data sets are anonymized (removal of a defined set of DICOM headers) after retrieval and before being forwarded for further processing within the P2D interface.

Since the PACS contains data with the same identifiers used in the hospital information system (HIS) and the DWH contains pseudonyms, the P2D interface approach requires the (de-)pseudonymization of DWH identifiers to and from PACS identifiers. Thus, the P2D provides a module that connects to the interface of an identity management server (managed by a trusted third party) to retrieve identifiers for a single or a list of pseudonyms of a patient, an Instance UID, or an Accession Number. Mappings between both identifier types are only used temporarily in the P2D interface for the time of the query. The P2D query interface does not release any identifying data. P2D currently provides a connector module to our self-developed identity management system but can be extended to support others.

The P2D interface provides a REST-style query interface that can be used to query the PACS with pseudonyms. A P2D query is defined by various HTTP GET/POST parameters, which includes the basic query type (bulk metadata or ad hoc) and further parameters: a list of identifiers (representing a patient, an image, a series, a study or an accession number), a time frame (from which the data is to be extracted), an image output format (JPEG or DICOM), a modality, an image selection method (all/first/middle image of a series), and the scope of the metadata to be extracted (basic/extended).

The bulk metadata extractor was designed to query a large number of patients in the PACS to insert/update DICOM metadata entries to the DWH database and is only to be used by technical DWH staff. It extracts header data from the DICOM images in a basic and an extended version to the DWH. While the basic extraction only enables to query the header data allowed by DICOM C-FIND (e.g., study/series/SOP instance UID, study and series description, modality, accession numbers), the extended extraction requires the download of the full DICOM data set and provides all headers. During DWH import, data from the DICOM information model (multiple images belong to a series which belongs to a study which belongs to a patient) needs to be mapped to the data model of a DWH (e.g., entity-attribute-value, EAV). Within the DWH, the data from the PACS is linked to the respective radiology report via the Accession Number and to all other clinical data via the patient and case. After metadata is extracted, the standard DWH query system can be used to query the variables by any DWH user.

The P2D ad hoc query system enables to query for metadata and DICOM images on demand from the PACS. Metadata can be queried for basic or extended headers and aggregated on the level of images, series, or studies. Its results are returned as CSV or JSON files. DICOM image queries are returned as JPEG or DCM files to a directory or provided as direct download.

**Fig. 2:**
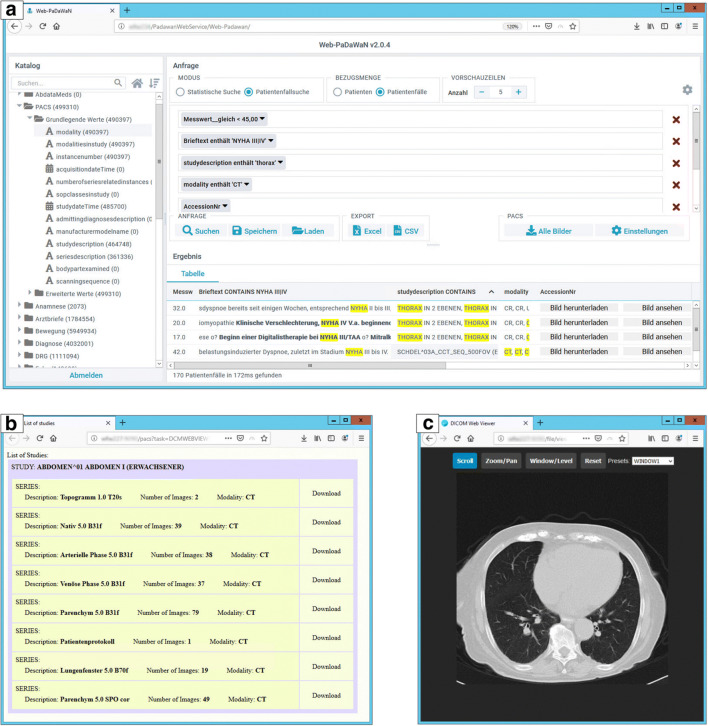
Screenshots of the PACS-to-DWH (P2D) integration. **a** An exemplary DWH query (top panel) with the catalog entries (left panel) of the PACS entries. Query results are shown on the bottom panel. The right-most columns present buttons to download (“Bild herunterladen”) or view images (“Bild ansehen”; results shown in **b**) of the patient or patient case. **b** The study/series list view of a single patient or patient case (depending on the DWH query). A click on the left part of the list leads to the view shown in **c**. A click on the right part provides a directory containing the downloaded images. **c** The view of a single DICOM CT series within the DICOM viewer

The DICOM viewer is provided by the HTTP file server and requires the ad hoc extractor to access images. After it has been started via the DWH, it lists all DICOM studies and their series of the patient or case with descriptions (cf. Fig. 2b). A click on a series leads either to the download of this series to a directory or to the DICOM image viewer (cf. Fig. 2c). We currently use the lightweight web-based DICOM viewer DWV.^4^

Per default, a standard user can only access anonymized descriptive statistics within our DWH. Access to the P2D ad hoc interface (required for image view and download) is only granted after consultation with the data protection officer and is ensured in the DWH by user authentication based on the hospital directory service. For potential audit, we store any query made by the P2D interface with username and time. In our setup, the user’s direct access to the P2D interface is deliberately limited. Access further requires the knowledge of a strong password, which is only shared with the DWH server. Any DWH query by a user involving the P2D interface results in the creation of a session with a temporary session key on the P2D engine including the patient identifiers involved in the query. Since the P2D ad hoc query system is used from the web-based DWH query interface, a technical user could get access to the temporary session key and could use the P2D directly. To provide DWH with control over query-able patients, this session key can only be used to query the patients in the session. Furthermore, we added a black/white-list of allowed modalities. For example, we have blocked any query with ultrasound (US) for image retrieval, because these images contain branded identifying texts within the pixel data (e.g., the patient name).

## Results

Figure 2 presents screenshots of the resulting integration of the P2D interface into the DWH user interface. Variables created by the bulk metadata extractor are shown in the catalog view (cf. Fig. 2a, left panel). An example query that uses data from the PACS and other clinical data is shown in the top panel and its results in the bottom panel (cf. first use case). Since the DWH user interface currently aggregates data by patient or patient case, an additional view is required to list associated DICOM studies and series (cf. Fig. 2b). Each entry can be selected for download or view. Alternatively, the download of all images associated to the whole query may be started after selecting required modalities. Since DICOM data often is large in size, it is downloaded to a server-side directory from where the researcher can retrieve the data. A sample view of a single image of a DICOM series is shown in Fig. 2c. The current web viewer provides very basic functions such as zoom, pan, and window level.

The query capabilities and performance of our DWH query system have already been presented elsewhere [26, 27, 28] and is similar if used with data extracted from a PACS. Thus, the next section focuses on the viability of extracting large amounts of data from the PACS to a DWH, which is done before a user has access to this data. The subsequent section focuses on the performance a user would experience while accessing the PACS ad hoc for image view or download.

### Bulk Metadata Extractor Performance

We tested the viability of extracting large amounts of metadata from the PACS via three approaches consisting of different degrees of granularity:

- Basic header/first image: the basic DICOM headers as provided by the DICOM C-FIND were extracted for the first image of each series (“first” refers to the attribute “Instance Number”),
- Basic header/all images: the basic DICOM headers were extracted for all patient images,
- Extended header/first image: all DICOM headers were extracted for the first image of each series after the images were retrieved by DICOM C-MOVE.

Out of the 1000 test patients, DICOM data of 988 patients were detected within the PACS. While targeting the first image of a series (approach a and c), about 26,000 single DICOM data sets (median (quartile0 of 10 (3–29) per patient) were retrieved, belonging to the same number of series (with a single image per series) and about 6400 studies (3 (1–8) per patient). If targeting all images (approach b), the number of retrieved data sets strongly increased. This resulted in about 990,000 single images (median (quartiles) of 103 (8–694) per patient), belonging to 26,000 series (10 (3–29) per patient) and 6400 studies (3 (1–8) per patient).

The data extraction of the test patients required an overall time and data usage of 45.3 min and 407 MiB for basic/first, 64.4 min and 406 MiB for basic/all, and 23.3 h and 117.4 GiB for extended/first. A full DICOM data set (i.e., a single image) had a median (quartiles) size of 566.4 (437.9–2355.9) KiB and required 2.032 (0.031–2.047) seconds to be downloaded from the PACS. In contrast, the pure C-FIND response had a size of 0.4 (0.4–0.4) KiB and required a fraction of a second to be downloaded.

The data within the PACS had some inconsistencies in the different extraction types, e.g., they were caused by DICOM files that were created externally. We did not handle any inconsistency yet, resulting in additional 5 (0.019%) images, which were not handled in the base version, and 69 (0.264%) images, which were not handled in the extended version.

Table 1 shows descriptive statistics of the extraction types aggregated per patient (more details are presented in supplement 1) using the production PACS. The data has a right-skewness leading to higher mean and maximum values (cf. Table 1). Consequently, the times and data rates required to extract a single patient may vary substantially. If only targeting the first image of a series much less DICOM data sets had to be downloaded (median of 10 versus 103 images per patient). When targeting the first image of a series, retrieving extended metadata took 139 times longer (in median) than retrieving basic metadata (12.52 versus 0.09 s per patient). The data transfer rates of the extended metadata extraction required 253 times the data rates of the basic variant (28.9 MiB versus 38.1 KiB). In comparison, the data collected using the Orthanc test setup shows a similar trend between the approaches (Table 2). However, there is an expected slight difference in the number of images per patient, based on differing patient selections. Further, the amount of data transferred from the PACS to the P2D is larger using Orthanc, which seems mainly based on a higher amount of data elements Orthanc includes into the DICOM response.

**Table 1:** Number of images, times, and data size required for the bulk metadata extraction of 1000 test patients from the production PACS, aggregated per patient (see supplementary tables for more details)

		Basic header		Extended header
		First image per series	All images	First image per series
Number of images per patient	Mean	26.5	1004	26.5
	Median (quartiles)	10 (3–30)	103 (8–693)	10 (3–30)
	Minimum	1	1	1
	Maximum	664	37,846	663
Time required to extract a patient’s data (seconds)	Mean	2.76	3.92	34.10
	Median (quartiles)	0.09 (0.03–2.25)	0.38 (0.03–2.67)	12.52 (4.11–37.30)
	Minimum	< 0.01	< 0.01	0.01
	Maximum	100.05	153.46	835.67
Data size to extract a patient’s data (KiB)	Mean	417.5	417.1	123,120.8
	Median (quartiles)	38.1 (3.0–278.1)	37.8 (3.0–277.2)	29,541.0 (6367.3–118,253.9)
	Minimum	0.2	0.2	0.2
	Maximum	15,577.1	15,577.1	3,939,238.9

**Table 2:** Number of images, times, and data size required for the bulk metadata extraction of 20 test patients from the Orthanc test setup, aggregated per patient (see supplementary tables for more details)

		Basic header		Extended header
		First image per series	All images	First image per series
Number of images per patient	Mean	44.1	1434.3	44.1
	Median (quartiles)	13 (5–67)	100 (32–1891)	13 (5–67)
	Minimum	1	1	1
	Maximum	171	7943	170
Time required to extract a patient’s data (seconds)	Mean	10.78	13.64	110.88
	Median (quartiles)	2.16 (1.08–9.46)	6.14 (0.78–29.76)	32.33 (9.19–147.75)
	Minimum	< 0.01	0.02	4.08
	Maximum	67.47	46.47	403.69
Data size to extract a patient’s data (KiB)	Mean	848.3	848.3	164,800.3
	Median (quartiles)	56.9 (13.9–1240.6)	56.9 (13.9–1240.6)	26,307.3 (4921.7–139,309.6)
	Minimum	0.7	0.7	0.2
	Maximum	4648.0	4648.0	1,863,962.9

An extrapolation can be used to inform on the actual times resource required to extract data of a whole PACS. We used the mean values from Table 1 for this estimation, as they are higher than the median and include outliers. The extraction of the basic headers of 500,000 patients would require about 16 days if targeting the first image and 23 days if targeting all images. The extraction of all headers and the first image per series would require 197 days.

### Ad Hoc Query Performance

While the metadata extraction must run before the data is usable via the DWH query interface, the ad hoc query may be used to query the PACS on demand. The queries required to create the list of a patient’s studies and series for the DICOM web viewer (cf. Fig. 2b) only requests few metadata from the PACS and required a median (quartiles) of 1.09 (1.03–1.14) seconds until shown to the user. Viewing a single DICOM series in the web viewer (cf. Fig. 2c) required to transfer full DICOM data sets to the browser, each with a separate request. Displaying a test series of 19 images required to transfer a mean 547.4 KiB per image, with the first image loaded after a median (quartiles) 5.55 (4.91–6.06) seconds and all images after 40.77 (38.60–41.63) seconds. A single image required a median of 2.15 s, which is comparable with the time spans the bulk extractor required for a single image (cf. online supplement). Downloading the same series to a directory required a median (quartiles) of 2.57 (2.57–2.79) seconds.

### Applied Use Cases

While the previous performance measurements described the viability of integrating the data from a PACS into a DWH, this section intends to exemplify the successful utilization of the P2D interface in four use cases. The queries were mainly carried out jointly by DWH and study staff.

1. To query the DWH using combinations of clinical and DICOM data to discover patient groups for studies: A very basic example of a query that considers clinical and DICOM data is shown in Fig. 2a. This query is used to select heart failure patients with a left ventricular ejection fraction (LVEF) value < 45% (value after information extraction from echocardiography report), a NYHA functional class of III or IV (narrative text search within discharge letters), DICOM data available from computer tomography (CT), and a DICOM study description containing the word “thorax.” After compiling the query within the DWH query interface using the variables previously created by the P2D bulk-metadata extractor, the system required 172 ms to preview results of 10 patients (image export using the ad hoc query not included). Such a query can be used for a feasibility analysis for a study that requires patients with heart failure and special radiological studies.
2. As an image data provider for clinical studies: A large clinical study conducted at our facility and already supported by our DWH required pseudonymized X-ray images of 592 patient cases. Input from the study staff was a list of patient pseudonyms each with the start/end date of the study-relevant hospitalization case. The study’s identity management system was used to map the study pseudonyms to the DWH patient identifiers. This list was then used to perform a P2D ad hoc query, which required about 40 min to download 1549 DICOM files (headers anonymized) into a temporary directory that was made available to the study staff.
3. To query and retrieve imaging and clinical data from the DWH for the training of neural networks: A prototype project was started for which patients with existing X-ray images and a specific finding in the radiology reports (using the DWH full-text search) were selected. Resulting DICOM images were exported. Associated clinical data, including radiology reports and information that links to the exported DICOM files, were exported for test patients as tabular files in order to annotate the X-ray images via information extraction algorithms for training purposes [29]. Image file content was anonymized and all other data pseudonymized.
  4.Extraction of metadata from a research PACS for analysis: An imaging research group at our facility uses a separate part of the clinical PACS as a research PACS. They required extractions of header data of a large number of DICOM data sets for analysis. Since common PACS are developed for clinical use, they do not provide such research-specific functionality. The researchers defined a list of subject pseudonyms, which were then used with the P2D interface to extract data of all headers ad hoc to text files (CSV or JSON). Individual DICOM headers of interest contained full-text documents and had to be further processed before analysis, i.e., the extraction of parameters from the text.

## Discussion

Here, we described the implementation of a P2D interface application that enables a complete production clinical PACS to be integrated into a pseudonymized clinical research DWH. We demonstrated our main objective (the viability of such an integration) and sub-objectives in terms of performance and application in selected use cases. Our system enables an important extension in functionality as it facilitates combinations of DICOM metadata and clinical data using potentially all hospital patients (cf. related work). It further provides ad hoc viewing and downloading of images while querying a clinical DWH of potentially all existing patients in the PACS.

The P2D metadata extractor serves as the basis to enable a DWH to query with combinations of clinical data and PACS DICOM metadata across all patients. This functionality was not yet in focus of related work [4, 13, 17, 20–22, 24]. We provide such query capabilities by extracting DICOM metadata of all patients from the PACS to the DWH, from where the query engine of a DWH can be used to search data. We evaluated different approaches to extract DICOM metadata from the PACS to a DWH, but not the query functionality of the DWH itself. Each approach is used to extract a different amount of metadata (basic versus extended data) for a different number of images (per image or per series), and requires specific times and data rates. The extraction times of another hospital’s PACS will likely differ, but the difference between the approaches will have a similar trend. The test on the Orthanc test setup also hints in this direction. Depending on the project need, the optimal approach can be chosen. A viable solution in terms of time, data rates, and resulting benefits could be the extraction of basic headers from single images per series for all patients and the extraction of extended headers for subgroups as needed for individual projects. The only alternative to such data extraction from the PACS would be the execution of ad hoc queries in the process of executing a DWH query. However, this would only provide parts of the functionality (search and extract of basic headers provided by C-FIND). Furthermore, it would be necessary to repeat individual PACS queries for each patient in each DWH query, which could affect the production availability of the PACS and thus patient care.

The P2D ad hoc query system allows to download and view all images of the PACS immediately after a DWH query. We demonstrated its performance by downloading and viewing a DICOM series from the web-based DICOM viewer. Related work projects also allow to download or view images, but usually not directly from a DWH query result table [24] or directly from the production PACS [20–23]. Our current web-based DICOM viewer is served with full DICOM files in order to provide interactive functionality to modify DICOM window levels. Our main interest in providing this viewer was to enable a manual selection of patients for a study after visual confirmation within the images. Such functionality might help to narrow the search for patients at an early stage and reduce the data that is to be downloaded and de-pseudonymized for a study. However, such a scenario would require an extension of the DWH to allow manual annotation of images while viewing the images, which we have not yet done. Accordingly, such application could not be tested in a real-world use case. Furthermore, the performance of the web-based viewer in manipulating DICOM files is considered not fast enough and requires further improvement.

We demonstrated the download of DICOM images with additional clinical data in use case 3. The image download is also based on the P2D ad hoc query system and was tested in several use cases. A main concern while using a functionality to download images directly from the PACS for a group of patients is the potentially large amount of images that might belong to a series or a study. For this reason, Murphy et al. [24] enabled to define a maximum number of studies per minute in order to prevent the i2b2 “server from flooding a PACS with requests creating a bottleneck situation or downtime”. Such a functionality is important and will be added to our system before providing the system to any DWH user.

The P2D interface allows basic and extended DICOM metadata to be extracted ad hoc from the PACS into tabular files for further analysis without the need to store entire images. This functionality still requires the retrieval of images from the PACS. However, these images do not need to be stored, which reduces required disk space. In addition, in some cases, the retrieval of a single image of a series may be sufficient, e.g., described in use case four, since many of the metadata of different images in a series are the same.

DICOM data is hierarchically structured in such a way that there is one study per examination that may contain several series, which in turn may contain several images. Our DWH is based on an entity-attribute-value (EAV) data model, which allows almost any data to be integrated and queried in a homogenized way. However, a standard EAV data model does not allow to create extensive relationships between individual data entries. We therefore extended our EAV model in order to enable the linkage of data on the level of a document (e.g., a laboratory analysis) and a group (e.g., attributes of a single laboratory value). In the current implementation, we link all metadata entries of a single DICOM image on the group level, which are further linked to the data entries of the radiology report on the document level. The study and series level is integrated as standard data entry but not part of the data model. Thus, analysis on those levels is currently only possible after data extraction from the DWH. We currently develop a connector to our DWH, but could easily provide one to any other DWH or data model.

However, while the data is provided with high detail and could be provided with the data’s original detail, querying such data in a simple way with any other data in a DWH is not trivial. The DWH query interface we provide to our users is optimized for a simple query and fast query capability [26] based on patient or patient case level aggregations [27]. The DWH can be used to query on the level of groups and documents, but not as easily as might be required for non-technical staff. Alternative data models might provide specific data models for specific data types, but might require adapted queries for each. More research is to be done on data models and their search functionalities to enable more simplified queries with multiple levels of aggregations and their combination.

During development, we observed differences in the extracted metadata of different extraction approaches. One source of errors was the EAV data model of our DWH. We used the study, series, and image identifier to calculate the group identifier of the EAV model. Our initial mapping resulted in collisions between multiple images, leading to overwriting of existing data. Another source of errors was the DICOM files originating from external hospitals. Their identifiers (patient, study, series, and image) within the DICOM headers were not reliably updated during the import procedure.

The P2D interface was developed as a pseudonymized front-end to a production PACS to support existing and planned research projects at our hospital. In order to connect a research DWH that contains pseudonymized data and a PACS that contains identifying data, a direct access to an identity management system is required. The connection between pseudonymized and identifying data only needs to be established temporarily on the P2D server side for the time needed to query the PACS and to pseudonymize extracted data. A DWH user will not see any of these identifiers, but the system still requires a strong access control. Of the modules provided by the P2D interface, the P2D ad hoc query (i.e., image viewer and downloader) is directly usable by a DWH user and might raise the greatest concern. Therefore, we have restricted it to staff authorized by the data protection officer. This process is documented within the hospital’s directory service and checked during DWH login using hospital credentials. Furthermore, the DWH creates a P2D session after login (if permitted and required), which can only be used to query the patients selected by the DWH query. The P2D metadata extractor on the other hand runs in the background and is not directly usable from the DWH query interface and thus might be the part of the system that could be used in any DWH environment. After having gained some experience with the system, we still use it with care, as not all exceptions in the DICOM data are obvious. An example is the documentation of identifying data (e.g., patient name) within the pixel data of ultrasound images.

The P2D provides interfaces to the PACS and to the DWH. The interface provided to the PACS is currently based on standard DICOM Message Exchange. With QIDO (Query based on ID for DICOM Objects) and WADO (Web Access to DICOM Objects), there are other parts of the DICOM standard that provide web-based access to a PACS. Both may simplify the connection between the PACS and the P2D. WADO may further allow to reduce the P2D’s bandwidth usage and increase its performance, since it allows to query special metadata without transferring the pixel data. However, it is not part of a minimal configuration of a usual production PACS and, thus, not reliably available. The pseudonymized interface the P2D offers to the DWH is currently based on a self-developed query language. Since QIDO and WADO are rather optimized for transactional data processing within hospitals, the P2D is rather optimized for bulk-access. Both do not provide the full functionality required by the P2D, e.g., bulk or ad hoc metadata extraction. However, a partial implementation of WADO would allow P2D to be used with other web-based DICOM viewers and might be added in the future.

While this work shows a way to unlock more data of a PACS for regular DWH queries, it still only allows to search via DICOM header data. Other approaches to search for imaging data will be based on the image content itself [26, 27], i.e., the pixel data. Such could for example be done by similarity-based search approaches [28] or image classification and object identification algorithms. However, the era of machine learning and artificial intelligence algorithms feeding back into medical research and clinical routine has just begun.

## Conclusion

A full integration of a production PACS with a research DWH is viable and enables various use cases in research. The combination of the extraction of basic DICOM metadata provided by C-FIND across the entire PACS and the extraction of extended metadata per subgroup and project may be the most viable way to enable a combined query with DWH metadata.

## Electronic supplementary material

ESM 1 (DOCX 23 kb)

## Footnotes

1 Orthanc DICOM server, Version 1.5.8: https://www.orthanc-server.com/ (last accessed: 28 February 2020)

2 DICOM toolkit (DCMTK), Version 3.6.4: http://dicom.offis.de (last accessed: 28 February 2020)

3 Defined in the DICOM Standard, Part 4, SOP Class Definitions

4 DWV (DICOM Web Viewer). https://github.com/ivmartel/dwv (last accessed: 28 February 2020)

## List of Abbreviations

CSV: comma-separated values
CT: computer tomography
DICOM: digital imaging and communications in medicine
DRG: diagnosis-related group
DWH: clinical data warehouse
EAV: entity-attribute-value
ID: identity
JSON: JavaScript Object Notation
KiB: kibibyte
LVEF: left ventricular ejection fraction
MiB: mebibyte
NYHA: New York Heart Association
P2D: PACS-to-DWH
PACS, Picture archiving and communication systems QIDO: Query based on ID for DICOM Objects
SOP: service-object pair
UID: unique identifiers
WADO: web access to DICOM objects
