# Supplementary material for: Unlocking the PACS DICOM Domain for its Use in Clinical Research Data Warehouses
Source: J Digit Imaging. 2020 Apr 20;33(4):1016–25. doi: 10.1007/s10278-020-00334-0 (PMC7522145; doi:10.1007/s10278-020-00334-0)
Supplement: Supplementary file 1 — (DOCX 23 kb) [file 10278_2020_334_MOESM1_ESM.docx]

Online Table 1. Number of images, times and data size required for the bulk metadata extraction of 1,000 test patients from the production PACS.

|  |  | Basic header | | Extended header |
| --- | --- | --- | --- | --- |
|  |  | First image per series | All images | First image per series |
| **A. Aggregated per DICOM image** | | | | |
| Time required to extract a SOP’s data [seconds] | Mean | 0.10 | <0.01 | 1.28 |
|  | Median (quartiles) | 0.00 (0.00-0.02) | 0.00 (0.00-0.05) | 2.03 (0.03-2.05) |
|  | Minimum | <0.01 | <0.01 | <0.01 |
|  | Maximum | 96.41 | 96.41 | 102.59 |
| Data size to extract a SOP’s data [KiB] | Mean | 0.4 | 0.4 | 4692.9 |
|  | Median (quartiles) | 0.4 (0.4-0.4) | 0.4 (0.4-0.4) | 566.4 (437.9-2355.9) |
|  | Minimum | 0.3 | 0.3 | 1.6 |
|  | Maximum | 0.5 | 0.5 | 1153276.8 |
| **B. Aggregated per DICOM series** | | | | |
| Number of Images per Series | Mean | 1.0 | 38.0 | 1.0 |
|  | Median (quartiles) | 1 (1-1) | 7 (1-38) | 1 (1-1) |
|  | Minimum | 1 | 1 | 1 |
|  | Maximum | 1 | 2400 | 1 |
| Time required to extract a series’ data [seconds] | Mean | 0.10 | 0.15 | 1.28 |
|  | Median (quartiles) | 0.00 (0.00-0.02) | 0.03 (0.00-0.14) | 2.03 (0.03-2.05) |
|  | Minimum | 0.00 | 0.00 | 0.00 |
|  | Maximum | 96.41 | 10.09 | 102.59 |
| Data size to extract a series’ data [KiB] | Mean | 15.9 | 16.0 | 4,714.4 |
|  | Median (quartiles) | 2.8 (0.4-15.6) | 2.8 (0.4-15.6) | 596.4 (452.9-2,358.7) |
|  | Minimum | 0.3 | 0.3 | 0.4 |
|  | Maximum | 1,031.1 | 1,031.1 | 1,153,277.5 |
| **C. Aggregated per DICOM study** | | | | |
| Number of Images per Study | Mean | 4.1 | 155.5 | 4.1 |
|  | Median (quartiles) | 2 (1-5) | 6 (2-47) | 2 (1-5) |
|  | Minimum | 1 | 1 | 1 |
|  | Maximum | 147 | 8,134 | 147 |
| Time required to extract a study’s data [seconds] | Mean | 0.43 | 0.61 | 5.25 |
|  | Median (quartiles) | 0.02 (0.00-0.05) | 0.02 (0.00-0.17) | 2.08 (2.05-6.23) |
|  | Minimum | 0.00 | 0.00 | 0.00 |
|  | Maximum | 96.49 | 35.25 | 160.51 |
| Data size to extract a study’s data [KiB] | Mean | 65.2 | 65.3 | 19,247.4 |
|  | Median (quartiles) | 2.1 (0.8-17.8) | 2.1 (0.8-17.9) | 5,613.1 (2,316.9-17,514.8) |
|  | Minimum | 0.3 | 0.3 | 3.9 |
|  | Maximum | 3,461.8 | 3,461.8 | 1,213,368.8 |

Online Table 2. Number of images, times and data size required for the bulk metadata extraction of 20 test patients from the Orthanc test setup.

|  |  | Basic header | | Extended header |
| --- | --- | --- | --- | --- |
|  |  | First image per series | All images | First image per series |
| **A. Aggregated per DICOM image** | | | | |
| Time required to extract a SOP’s data [seconds] | Mean | 0.16 | 0.01 | 1.70 |
|  | Median (quartiles) | 0.00 (0.00-0.02) | 0.00 (0.00-0.01) | 2.03 (0.03-2.05) |
|  | Minimum | <0.01 | <0.01 | <0.01 |
|  | Maximum | 32.89 | 37.60 | 100.78 |
| Data size to extract a SOP’s data [KiB] | Mean | 0.6 | 0.6 | 4,118.1 |
|  | Median (quartiles) | 0.6 (0.6-0.6) | 0.6 (0.6-0.6) | 548.4 (341.3-2,308.5) |
|  | Minimum | 0.4 | 0.4 | 4.2 |
|  | Maximum | 0.6 | 0.6 | 106,745.8 |
| **B. Aggregated per DICOM series** | | | | |
| Number of Images per Series | Mean | 1.0 | 33.3 | 1.0 |
|  | Median (quartiles) | 1 (1-1) | 11 (1-35) | 1 (1-1) |
|  | Minimum | 1 | 1 | 1 |
|  | Maximum | 1 | 822 | 1 |
| Time required to extract a series’ data [seconds] | Mean | 0.26 | 0.33 | 1.77 |
|  | Median (quartiles) | 0.00 (0.00-0.02) | 0.06 (0.01-0.24) | 2.03 (0.03-2.05) |
|  | Minimum | <0.01 | <0.01 | <0.01 |
|  | Maximum | 61.02 | 37.60 | 100.78 |
| Data size to extract a series’ data [KiB] | Mean | 38.4 | 38.4 | 7,457.6 |
|  | Median (quartiles) | 11.7 (1.1-40.1) | 11.7 (1.1-40.1) | 1,131.1 (666.8-4,747.8) |
|  | Minimum | 0.9 | 0.9 | 4.8 |
|  | Maximum | 1,030.3 | 1,030.3 | 172,123.2 |
| **C. Aggregated per DICOM study** | | | | |
| Number of Images per Study | Mean | 3.8 | 130.8 | 3.7 |
|  | Median (quartiles) | 2 (1-4) | 3 (2-34) | 1 (1-3) |
|  | Minimum | 1 | 1 | 1 |
|  | Maximum | 28 | 2,935 | 33 |
| Time required to extract a study’s data [seconds] | Mean | 0.94 | 1.32 | 6.99 |
|  | Median (quartiles) | 0.02 (0.00-0.03) | 0.02 (0.00-0.31) | 2.14 (2.03-6.17) |
|  | Minimum | <0.01 | <0.01 | <0.01 |
|  | Maximum | 61.05 | 37.60 | 121.98 |
| Data size to extract a study’s data [KiB] | Mean | 151.3 | 151.3 | 29,353.6 |
|  | Median (quartiles) | 3.2 (2.0-35.8) | 3.2 (2.0-35.8) | 16,712.7 (4,720.1-35,860.1) |
|  | Minimum | 0.9 | 0.9 | 55.2 |
|  | Maximum | 3,432.3 | 3,432.3 | 315,665.4 |
